# Supplementary material for: Understanding the Clinical Significance of MUC5AC in Biliary Tract Cancers
Source: Cancers (Basel). 2023 Jan 9;15(2):433. doi: 10.3390/cancers15020433 (PMC9856870; doi:10.3390/cancers15020433)
Supplement: Supplementary file 1 [file cancers-15-00433-s001.zip › cancers-2137989-supplementary.pdf]

Supplementary Materials

Understanding the Clinical Significance of MUC5AC in Biliary Tract Cancers

Katherine K. Benson, Ankur Sheel, Shafia Rahman, Ashwini Esnakula and Ashish Manne*

Table S1. MUC5AC Antibody Variants, Domains, and Localization in Biliary Tissues

Antibody Name	Immature or Mature MUC5AC?	Antibody Type	Domain of MUC5AC Protein	Binding Site(s) in Normal Biliary Tissues and/or BTC Tissues	Reference(s)
CLH2	Immature	Mouse mAb	Core tandem repeat	Cytoplasm	[1,2]
M5P-b1		Chicken and rabbit pAb	Core tandem repeat	Cytoplasm	[1,3]
MAN-5ACI	Mature	Rabbit pAb	Tandem repeat region & C-terminal peptide	Cytoplaasm, apical surface, lumen of tumor glands, and nearby stromal tissues	[1,4]
Lum5-1 EU-batch		Rabbit pAb	Tandem repeat region & C-terminal peptide	Not described	[1,5]
21M1		Mouse mAb	C-terminal region	Not described	[1,5,6]
45M1		Mouse mAb	C-terminal region	Cytoplasm, lumen of tumor glands, and nearby stromal tissues	[2,7]
S121		Mouse mAb	Carbohydrate-associated antigen on MUC5AC	Cytoplasm, apical surface, and some nearby Kupffer/inflammatory cells	[8]
mAB-22C5		Mouse mAb	Not described	Partially described (includes biliary mucus of tumors)	[9]
CA-S27		Mouse mAb	Lewis-a (Le^a) associated glycan moiety on MUC5AC	Not described	[10]

References

1. Krishn, S.R.; Ganguly, K.; Kaur, S.; Batra, S.K. Ramifications of secreted mucin MUC5AC in malignant journey: A holistic view. *Carcinogenesis* **2018**, *39*, 633–651. https://doi.org/10.1093/carcin/bgy019.
2. Aishima, S.; Kuroda, Y.; Nishihara, Y.; Taguchi, K.; Taketomi, A.; Maehara, Y.; Tsuneyoshi, M. Gastric mucin phenotype defines tumour progression and prognosis of intrahepatic cholangiocarcinoma: Gastric foveolar type is associated with aggressive tumour behaviour. *Histopathology* **2006**, *49*, 35–44. https://doi.org/10.1111/j.1365-2559.2006.02414.x.
3. Sasaki, M.; Nakanuma, Y.; Kim, Y.S. Expression of apomucins in the intrahepatic biliary tree in hepatolithiasis differs from that in normal liver and extrahepatic biliary obstruction. *Hepatology* **1998**, *27*, 54–61. https://doi.org/10.1002/hep.510270110.

4. Wongkham, S.; Sheehan, J.K.; Boonla, C.; Patrakitkomjorn, S.; Howard, M.; Kirkham, S.; Sripa, B.; Wongkham, C.; Bhudhisawasdi, V. Serum MUC5AC mucin as a potential marker for cholangiocarcinoma. *Cancer Lett* **2003**, *195*, 93–99. https://doi.org/10.1016/s0304-3835(02)00691-2.
5. Matull, W.R.; Andreola, F.; Loh, A.; Adiguzel, Z.; Deheragoda, M.; Qureshi, U.; Batra, S.K.; Swallow, D.M.; Pereira, S.P. MUC4 and MUC5AC are highly specific tumour-associated mucins in biliary tract cancer. *Br J Cancer* **2008**, *98*, 1675–1681. https://doi.org/10.1038/sj.bjc.6604364.
6. Bara, J.; Gautier, R.; Mouradian, P.; Decaens, C.; Daher, N. Oncofetal mucin M1 epitope family: Characterization and expression during colonic carcinogenesis. *Int J Cancer* **1991**, *47*, 304–310. https://doi.org/10.1002/ijc.2910470222.
7. Lidell, M.E.; Bara, J.; Hansson, G.C. Mapping of the 45M1 epitope to the C-terminal cysteine-rich part of the human MUC5AC mucin. *Febs j* **2008**, *275*, 481–489. https://doi.org/10.1111/j.1742-4658.2007.06215.x.
8. Silsirivanit, A.; Araki, N.; Wongkham, C.; Pairojkul, C.; Narimatsu, Y.; Kuwahara, K.; Narimatsu, H.; Wongkham, S.; Sakaguchi, N. A novel serum carbohydrate marker on mucin 5AC: Values for diagnostic and prognostic indicators for cholangiocarcinoma. *Cancer* **2011**, *117*, 3393–3403. https://doi.org/10.1002/cncr.25912.
9. Bamrungphon, W.; Prempracha, N.; Bunchu, N.; Rangdaeng, S.; Sandhu, T.; Srisukho, S.; Boonla, C.; Wongkham, S. A new mucin antibody/enzyme-linked lectin-sandwich assay of serum MUC5AC mucin for the diagnosis of cholangiocarcinoma. *Cancer Lett* **2007**, *247*, 301–308. https://doi.org/10.1016/j.canlet.2006.05.007.
10. Silsirivanit, A.; Araki, N.; Wongkham, C.; Vaeteewoottacharn, K.; Pairojkul, C.; Kuwahara, K.; Narimatsu, Y.; Sawaki, H.; Narimatsu, H.; Okada, S.; et al. aa-S27: A novel Lewis a associated carbohydrate epitope is diagnostic and prognostic for cholangiocarcinoma. *Cancer Sci* **2013**, *104*, 1278–1284. https://doi.org/10.1111/cas.12222.
